# Deficiency of the *Tbc1d21* gene causes male infertility with morphological abnormalities of the sperm mitochondria and flagellum in mice

**DOI:** 10.1371/journal.pgen.1009020

**Published:** 2020-09-25

**Authors:** Ya-Yun Wang, Chih-Chun Ke, Yen-Lin Chen, Yu-Hua Lin, I-Shing Yu, Wei-Chi Ku, Moira K. O’Bryan, Ying-Hung Lin

**Affiliations:** 1 Graduate Institute of Biomedical and Pharmaceutical Science, Fu Jen Catholic University, New Taipei City, Taiwan; 2 PhD Program in Nutrition & Food science, Fu Jen Catholic University, New Taipei City, Taiwan; 3 Department of Urology, En Chu Kong Hospital, New Taipei City, Taiwan; 4 Department of Pathology, Cardinal Tien Hospital, New Taipei City, Taiwan; 5 School of Medicine, Fu Jen Catholic University, New Taipei City, Taiwan; 6 Division of Urology, Department of Surgery, Cardinal Tien Hospital, New Taipei City, Taiwan; 7 Department of Chemistry, Fu Jen Catholic University, New Taipei City, Taiwan; 8 Laboratory Animal Center, College of Medicine, National Taiwan University, Taipei, Taiwan; 9 School of Biological Sciences, Monash University, Melbourne, Victoria, Australia; HudsonAlpha Institute for Biotechnology, UNITED STATES

## Abstract

Approximately 2–15% of couples experience infertility, and around half of these cases are attributed to male infertility. We previously identified *TBC1D21* as a sterility-related RabGAP gene derived from infertile men. However, the *in vivo* function of TBC1D21 in male fertility remains unclear. Here, we show that loss of *Tbc1d21* in mice resulted in male infertility, characterized by defects in sperm tail structure and diminished sperm motility. The mitochondria of the sperm-tail had an abnormal irregular arrangement, abnormal diameter, and structural defects. Moreover, the axoneme structure of sperm tails was severely disturbed. Several TBC1D21 interactors were selected via proteomic analysis and functional grouping. Two of the candidate interactors, a subunit protein of translocase in the outer membrane of mitochondria (TOMM20) and an inner arm component of the sperm tail axoneme (Dynein Heavy chain 7, DNAH7), confirmed *in vivo* physical co-localization with TBC1D21. In addition, TOMM20 and DNAH7 detached and dispersed outside the axoneme in *Tbc1d21-*deficient sperm, instead of aligning with the axoneme. From a clinical perspective, the transcript levels of *TBC1D21* in sperm from teratozoospermia cases were significantly reduced when compared with those in normozoospermia. We concluded that TBC1D21 is critical for mitochondrial and axoneme development of mammalian sperm.

## Author summary

Male sterility affects one in seven couples, and around half of these are the result of male factor disorders. The common conditions of male infertility cases include reduced sperm motility, low sperm account, and spermatozoa with abnormal morphology. We previously identified TBC1D21 as a sterility-related RabGAP gene from infertile men. However, the in vivo function of TBC1D21 in male fertility remains untested. Here, we created *Tbc1d21* knockout mice. The knockout males were completely sterile, and had sperm-tail and mitochondria defects. Two interactors of TBC1D21, TOMM20, and DNAH7, may account for the irregular and defective patterns observed in *Tbc1d21*^-/-^ mouse sperm tails. From a clinical perspective, *TBC1D21* transcripts are reduced in sperm from patients exhibiting teratozoospermia. Our results indicate that TBC1D21 plays a critical role in the formation of intact mitochondria, and structure of mammalian sperm.

## Introduction

Epidemiological studies reveal that 2–15% of couples are affected by decreased fertility globally, with approximately half of the cases relating to male causes of infertility [1,2]. Male sterility is a multifactorial disease with many causes, including anatomic abnormalities, gametogenesis defects, immunological problems, endocrine dysregulations, ejaculatory defects, environmental toxic exposures, and genetic alterations [3]. Genetic abnormalities are known to account for 15–30% of cases, although this number is likely to be considerably underestimated [4,5]. Notably, the molecular functions of many spermatogenic genes remain unknown *in vivo* [6,7].

The major events of sperm generation include mitosis of spermatogonia, meiosis of spermatocytes, morphological differentiation of haploid gametes during spermiogenesis, maturation of spermatozoa in the epididymis, and, within the female reproductive tract, capacitation, the acrosome reaction, and fertilization [8,9]. The processes of mammalian spermiogenesis are comprised of four phases [10,11]: (1) during the Golgi phase, a Golgi-derived acrosomal vesicle is formed and attaches to the nuclear envelope in round spermatids; (2) during the cap phase, the acrosomal vesicle enlarges and spreads to cover the anterior pole of the sperm head, and nuclear contents become more condensed; (3) the acrosome phase wherein a) the manchette forms, which ultimately plays a role in shaping the posterior half of the sperm head and as a protein transport network; b) the development and elaboration of the flagellum occurs, including the axoneme, the outer dense fibers, and the fibrous sheath; and c) mitochondria are recruited into the mitochondrial sheath defining the midpiece; and (4) as a final maturation phase, the residual cytoplasm of the sperm is removed by Sertoli cells, and sperm are released in a process called spermiation. The net results of these processes are spermatozoa, which have a highly condensed haploid genome, an apical acrosome, and a flagellum, including a midpiece comprising helically arranged mitochondria.

The small GTP-binding protein superfamily is comprised of a range of sub-family members, including Rab, CDC42, Ras, Rho, Ran, Rap, Ral, and Rhe [12]. Small GTP-binding proteins play essential roles in a variety of cellular pathways and functions, including secretion, intracellular vesicular traffic, cytoskeleton remodeling, cell polarity, survival, apoptosis, and cell proliferation [12]. Within the category of small GTP-binding protein regulators, the guanine nucleotide exchange factors (GEFs) and GTPase-activating proteins (GAPs) are major modulators of small GTP-binding protein activity [13,14]. GEFs catalyze the dissociation of GDP-binding of small GTP-binding proteins and, thus, the GDP is released and subsequently replaced by GTP. GAPs activate the GTPase function of small GTP-binding proteins, promoting the conversion of GTP to GDP, thus, assisting inactivation. The accurate spatio-temporal switching between small GTP-binding protein activation and inactivation is vital for cellular fates. Recently, dysregulation of small GTP-binding proteins and their modulators has been shown to cause various conditions, including cancer, endocrine diseases, and male infertility [15].

Different families of small GTP-binding proteins are regulated by specific GAP proteins. In mammals, a large portion of RabGAP proteins have a Tre-2/Bub2/Cdc16 (TBC) domain of approximately 200 amino acids [16–18]. The TBC domain originates from the Tre-2 oncogene and the yeast cell cycle regulators BUB2 and CDC16, which share a high degree of homology and possess a GTPase-activating function for the specific Rab proteins [16]. Thus far, over 40 proteins have been characterized as RabGAP proteins in mice and humans. More specifically, at least 10 Rab proteins have been implicated in the acrosomal reaction, sperm head and tail formation, fertilization, or intracellular trafficking [19–29]. In our previous study, TBC1D21 was identified through a cDNA microarray. Its levels were downregulated within testicular tissue from the Sertoli cell-only syndrome cases, when compared with fertile men [30]. It displays a high testis-enriched expression pattern, and TBC1D21 localization is restricted to the peri-acrosomal and manchette regions during the cap and acrosome phase of spermiogenesis [31]. In addition, TBC1D21 is localized within the mature sperm tail. Moreover, TBC1D21 has GTPase-activating activity and interacts with proteins, including RAB10, RAB5C, and RAP1, as identified through co-immunoprecipitation (co-*IP*) and nano-liquid chromatography-tandem mass spectrometry (nano-LC–MS/MS) analysis [32]. The *in vivo* function of TBC1D21, however, remains untested. In the present study, we generated *Tbc1d21* knockout mice to evaluate the role of TBC1D21 in the male reproductive function *in vivo*. Herein we reveal that TBC1D21 plays an essential role in maintaining the sperm-tail function and, thus, male fertility.

## Materials and methods

### Ethics statement

The animal experiments were approved by the Fu Jen Laboratory Animal Care and Use Committee (No: A10180, Date of approval: 05/07/2013; No: A10724, Date of approval: 08/ 01/2018).

### Generation of *Tbc1d21* knockout mice

A murine genomic fragment carrying the entire *Tbc1d21* locus was constructed in the BAC bMQ374D12 clone (Sanger institute, UK), subcloned in pL253, and inserted into the loxp sites (S1A Fig). It was used for replacing the wild-type (WT) allele of *Tbc1d21* in C57BL/6 mouse embryonic stem cells (MESCs). MESCs cell clones bearing the *loxp* site allele were identified by Southern blotting. The clones were isolated and injected into the C57BL/6J blastocysts for the production of chimeric mice. The chimeric male mice were mated with *Sox2-Cre* transgenic female mice (Jackson Laboratory, Stock No: 008454, Bar Harbor, ME, USA), and the pups exhibited Cre recombinase activity at the epiblast stage on the embryonic day 6.5. Pups were genotyped by extracting genomic DNA from the tail and analysis by PCR. The primers used were as follows: CU (5'- ATAGCGGCCGCAGAAGCTGTGTTCAGC CTC -3'), FD (5′- ATAGTCGACCACTTAACCGAATACCTGC -3′) and JD (5′- ATAGTCGAC CGCCATCTTGG AATC AG -3′). Food and water were provided freely, and standard care was offered based on international and standard ethical guidelines. Mice were humanely killed at 3 months of age by anaesthesia with isoflurane. The testes and epididymides were collected and weighed (>6 mice of each genotype). The reproductive ability tests of each group of mice were compared between pups from the same pregnancy.

### Primary antibodies used in the study

The antibodies used in this study are were: SEPT7 antibody (sc-20620, Santa Cruz), Basigin antibody (GTX62657, GeneTex), FLAG antibody (F1804, Sigma), GFP antibody (Abcam, ab290), TOMM20 antibody (11802-1-AP, Proteintech), TBC1D21 antibody (ab119060, Abcam), and anti-α-tubulin antibody (GTX628802, GeneTex).

### Histological evaluation and immunofluorescence assay

WT and *Tbc1d21*^*-/-*^ mice were sacrificed at 3 months of age, and their epididymides, testes, and sperm were collected. Epididymides and sperm were fixed in PBS with 4% paraformaldehyde, and the testes were fixed in the Bouin's solution (Sigma-Aldrich) then embedded with paraffin wax using standard methods. Sections of these paraffin-embedded tissues were stained with haematoxylin and eosin (H&E) for histological evaluation. For sperm immunofluorescence (IF) assay, non-specific antibody binding was minimised by incubating sperm sections were blocked with Blocking solutions (Dako). Sections were incubated overnight at 4°C with diluted primary antibodies. Primary antibody binding was detected using secondary antibodies conjugated with Alexa Fluor 405, Alexa Flour 488, or Alexa Fluor 568 were used. The midpiece was stained by Mito-tracker (Invitrogen, cat no. M22425), followed by DAPI staining, and mounted with the Dako mounting medium.

### Sperm analysis

The epididymides and vas deferens isolated from 3-month-old mice were cut into small pieces in 2 ml of the M16 medium (Sigma-Aldrich) and sperm allowed to swim into the medium, which was maintained at 37°C and 5% CO_2_. After 30 min, the medium was collected and diluted for sperm number and motility assessment. For evaluating sperm motility, sperm samples (sperm number>200) were used. “Progressive motility” of sperms is characterized by “spermatozoa moving actively, either linearly or in a large circle, regardless of speed” [33]. For evaluating the sperm morphology, the sperm were collected through centrifugation at 300× *g* for 5 min and were then resuspended in PBS. Sperm were spread onto coated slides and air-dried. One hundred spermatozoa per mouse were examined per group through staining with Mito-tracker and DAPI under a microscope at 400X magnification. All results were obtained from experiments performed on at least 6 mice per genotype group and 100 sperm per mouse. Data were analysed using the Student's *t* test to determine significant differences between the two groups. Error bars have been presented as the mean ± SEM. A p-value of *<*0.05 was considered statistically significant.

### Electron microscopy

All protocols were used as utilized in our previous study [34]. The sperm were collected from the epididymides and vas deferens from 3-month-old mice and were immediately fixed with 4% paraformaldehyde and 0.1% glutaraldehyde overnight at 4°C. Sperm were then rinsed with 0.1 M phosphate buffer (pH 7.2) and treated with 1% osmium tetroxide at room temperature for 2 hours. After washing with phosphate buffer again, the sperm were slowly dehydrated by increasing the percentage of ethanol. Sperm were then embedded using the Spurr’s resin kit (cat-14300; EMS) at room temperature, overnight. Embedded tissues were cut into 75-nm thick sections and placed on copper grids and ultramicrographs were acquired via using a transmission electron microscope (JEM-1400; JEOL) at 100 Kva.

### Measurement of sperm ATP levels in vitro

An average of 5 × 10^6^ sperm was used for the ATP measurement experiment. The experiment was performed according to the manufacturer's instructions (ATP assay kit, ab83355; Abcam). Briefly, the sperm samples were washed twice with PBS, resuspended in ATP assay buffer, vortexed, and placed on ice for 15 min. After incubation, the lysate was centrifuged and the supernatant was transferred to a fresh tube. The supernatant was mixed with the reaction reagent and incubated at room temperature for 30 min. Lastly, the ATP level was determined fluorometrically using a luminometer (Infinite M200 Pro; Tecan Life Sciences).

### Co-immunoprecipitation analysis

NTERA-2 d.D1 (NT2D1, a pluripotent human testicular embryonal carcinoma cell line) was used for the Co-IP experiment. The co-immunoprecipitation analysis (Co-IP) analysis was performed according to our previous study [35,36]. The pFLAG-Tbc1d21 plasmids were transfected into the cells. The cell lysates (4 mg protein in 1 mL) were pre-cleared by incubating with A/G beads (Santa Cruz Biotechnology)) for 1 h at 4°C on a rotator. Following, the clear supernatant was incubated overnight with the control IgG or anti-FLAG (Sigma-Aldrich), respectively, and with A/G beads (Santa Cruz Biotechnology)). The samples were then washed twice with 1X PBS, followed by immunoblotting (IB). The following antibodies were used for IB: anti-FLAG, anti-RAB10, anti-TOMM20, and anti-GFP antibody. Western blot analysis was performed according to the standard protocol as used in our previous study [36].

### DuoLink proximity ligation assay

Duolink proximity ligation assays (Duolink PLA) were performed according the manufacturer’s instructions (Sigma-Aldrich) and our previous study [37]. Concisely, purified murine sperm were fixed on the slides, following incubation by Duolink blocking solution for 60 minutes at 37°C. Anti-TBC1D21 and anti-TOMM20 or anti-DNAH7 antibodies were diluted by Duolink antibody diluent and incubated 12 hours at 4°C. After washing out the primary antibody twice with 1X wash buffer A, Duolink PLA probes PLUS (mouse) and MINUS (rabbit) were mixed in Duolink antibody diluent and added on the slides for 60 minutes at 37°C. After washing out the Duolink PLA probes by 1X wash buffer A, Duolink ligation was performed (30 minutes at 37°C). If the two PLA probes were less than 40 nanometers from one another, the binding and ligation would occur. Following the washing step, amplification and hybridization with fluorescently labeled nucleotides was accomplished using Duolink amplification buffer and polymerase (100 minutes at 37°C). Midpieces were stained using the Mito-tracker.

### Data mining of TBC1D21 expression in human testis and teratozoospermia cases

The figures of TBC1D21 localization in human testicular tissues were collected from The HUMAN PROTEIN ATLAS (https://www.proteinatlas.org/ENSG00000167139-TBC1D21/tissue/testis#img). The profiles of *TBC1D21* transcripts in human teratozoospermia were obtained from the published GEO set (GSE6872; www.omicsdi.org/dataset/geo/GSE6872) generated using the GPL570 Affymetrix Human Genome U133 Plus 2.0 Array platform. Platts *et al* collected 13 semen samples from normal fertile individuals who had previously fathered a child through natural conception and 8 semen samples from patients with severe tetrazoospermia, which contained ≤ 3% sperm with normal morphological characteristics; there were no other abnormal semen parameters in the patients. The sperm in samples collected from 6 of 8 patients with severe tetrazoospermia exhibited midpiece or tail defects [38]. The values were normalized to those of mean gene expression levels and analyzed using an unpaired *t-test*.

## Results

### TBC1D21 is essential for male fertility in mice

To dissect the *in vivo* function of *Tbc1d21* in male reproduction, we knocked out the *Tbc1d21* locus in mice. The targeting vector was designed to replace exons 4–11 of *Tbc1d21* with a *neo* cassette (S1A Fig). Successful replacement of the wild-type allele by the inserted allele in MESCs was confirmed by Southern blotting (S1A Fig, right upper). The chimeric mice, generated by injecting *Tbc1d21*^+/-^ MESCs into C57BL/6 blastocysts, were mated with transgenic mice harboring the cre recombinase (cre)-coding sequences, expressed under the *Sox2*-promoter. Over 100 mice were generated and genotyped using specific primer pairs (CU+ JD and CU+FD) (S1A Fig, lower right). First, the ratio of genotypes of the mice were close to Mendelian rules (+/+: +/-:-/- = 1:2:1), indicating that TBC1D21 does not play a critical role during embryogenesis. Second, testes, epididymis, and vas deferens weights of *Tbc1d21*^-/-^ mice are comparable with wild-type male mice (S1B Fig). Third, the histology of testis sections appeared qualitatively normal (S1C and S1D Fig). In contrast, the levels of sperm in the epididymes and vas deferens of *Tbc1d21*^-/-^ mice reduced significantly compared to those in wild-type mice, and the knockout mice were completely sterile when mated with wild-type female mice (Table 1). In addition, the knockout female were fertile. Collectively, these data reveal that TBC1D1 is essential for the establishment of male fertility, and required for both the production of normal sperm number and sperm function.

**Table 1:** Targeted disruption of *Tbc1d21* induces male infertility in mice.

Mating genotype		Average number of pups per litter^a^
Male	Female	
*Tbc1d21*^*+/+*^ *× Tbc1d21*^*+/+*^		8.00 ± 0.76
*Tbc1d21*^*–/–*^*× Tbc1d21*^*+/+*^		0^b^

a Values are expressed in terms of mean ± SEM from 8 mice.

b ***p < 0.0001, analyzed using Student's *t* test.

### TBC1D21 is required for normal sperm formation

**Fig 1:**
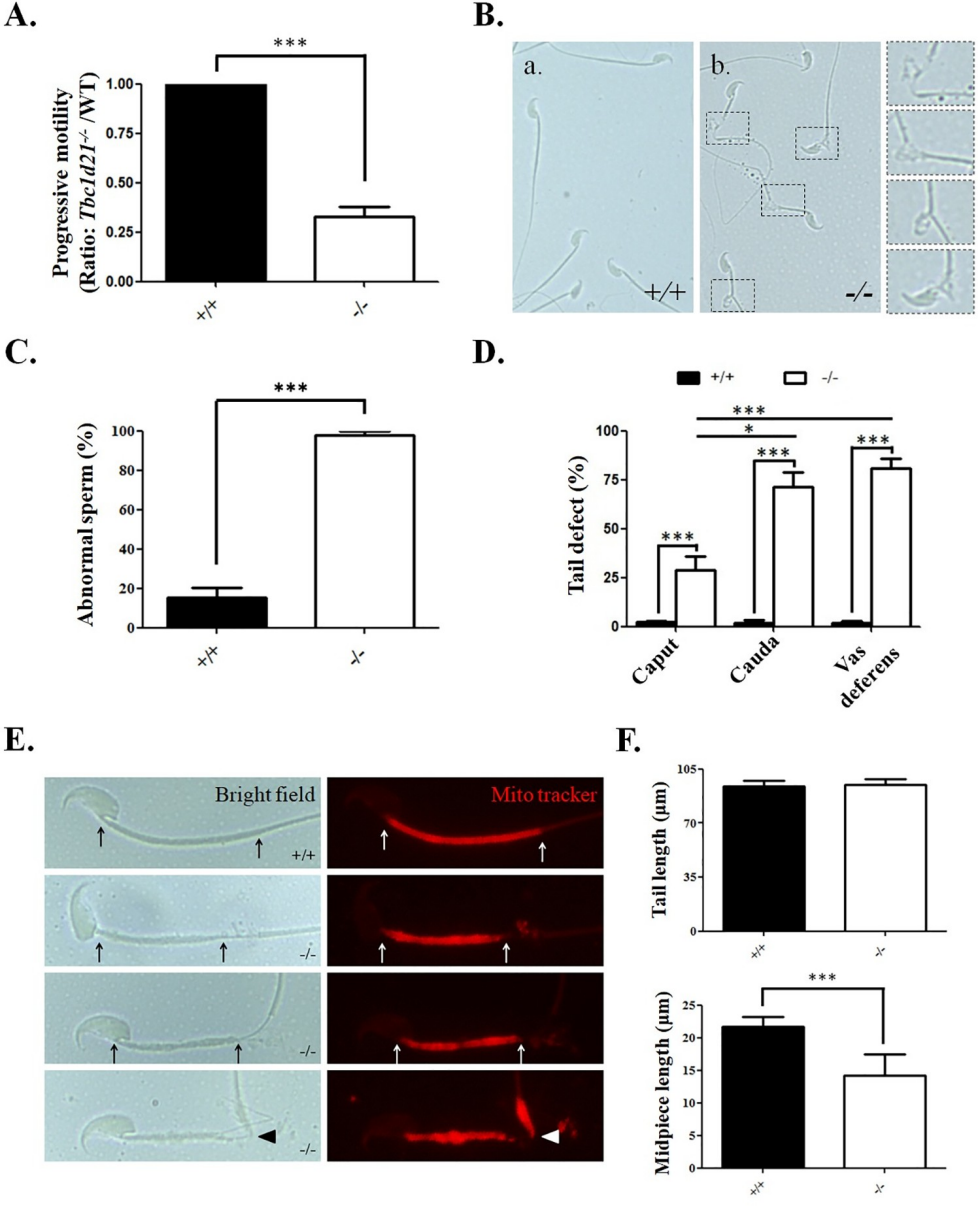
Knockout of *Tbc1d21* in mice causes sperm tail defects. (A) Sperm collected from 2-month-old wild-type and *Tbc1d21-*deficient mice. Analysis of the ratio of sperm with progressive motility in *Tbc1d21*^*-/-*^ mice, which were normalized to the wild-type mice to calculate the fold change. (B) Sperm collected from *Tbc1d21*^*-/-*^ mice had a defective tail (b), compared with the wild-type (a). Enlarged figures of the defective tail at the left panel represents the back-dot square of (b). (C) The statistical analysis showing the ratio of abnormal sperm isolated from wild-type and *Tbc1d21*^*-/-*^ mice. (D) Statistical analysis representing the percentage of tail-defective sperm, collected from the caput of the epididymis, cauda of the epididymis, and the vas deferens. (E) The figures show the disrupted morphology of the midpiece region (between the arrows or arrowhead indicated) of *Tbc1d21* deficient sperm compared to sperm from wild-type mice (Left panel: Bright filed; Right panel: staining with Mito-tracker). (F) The statistical analysis represents the length of the sperm tail and midpiece of wild-type and *Tbc1d21*^*-/-*^ mice. Each bar represents the mean ± standard error of the mean (SEM); Mice number (n = 3) per genotype. Sperm number >100 per mice. *Significant differences compared with the wild-type mice (***p < 0.0001, analysed using Student's *t* test).

In order to precisely define the effects of loss of TBC1D21 on reproductive function, we analyzed sperm motility using microscopic analysis. Sperm from *Tbc1d21*^-/-^ mice had significantly compromised ability for progressive motility (Fig 1A). Similarly, the sperm from *Tbc1d21*^-/-^ mice displayed abnormal morphology (98.03 ± 0.65% vs. 15.43 ± 1.88%) when compared to the wild-type (Fig 1B and 1C.). Moreover, the major defects were found at the sperm tail, which showed obvious breakage (54.62 ± 2.67% vs. 0.46 ± 0.17%). According to previous studies, there are two possible pathological reasons for tail defects: (a) Abnormal terminal differentiation of spermatids occurred within the testes; (b) sperm tails were damaged during transportation from the epididymis to the vas deferens. To test these possibilities, sperm were isolated from (i) the caput of epididymis, (ii) the cauda of the epididymis, and (iii) the vas deferens during sperm transport, and were evaluated. Fig 1D shows that the percentages of sperm with tail defects in the caput of epididymis of *Tbc1d21*^-/-^ mice were higher compared to those of the wild-type (Lane 1). During the transport of sperm from the caput of the epididymis to the vas deferens, the percentages of tail-defected sperm in *Tbc1d21*^-/-^ mice increased when compared to the caput (Lanes 2 and 3). Collectively, these data suggest that Tbc1d21-/- sperm defects originated during spermatogenesis, but were exacerbated during epididymal maturation.

To define the defective regions of *Tbc1d21*^-/-^ sperm tails, the sperm were stained with a Mito-tracker to label mitochondria. Image analysis revealed that sperm from *Tbc1d21*^-/-^ had a severely disrupted midpiece region (Fig 1E). Specifically, the midpiece was noticeably shorter, and in places mitochondria were missing or present as thickened layer (Fig 1F). Based on these data, we hypothesized that TBC1D21 is involved in the transportation, loading, or adhesion of mitochondria into the midpiece of sperm. Collectively the major phenotype observed in *Tbc1d21*^-/-^ males is consistent with teratozoospermia in humans.

### TBC1D21 is dispensable for the formation of the annulus structure of the sperm tail

**Fig 2:**
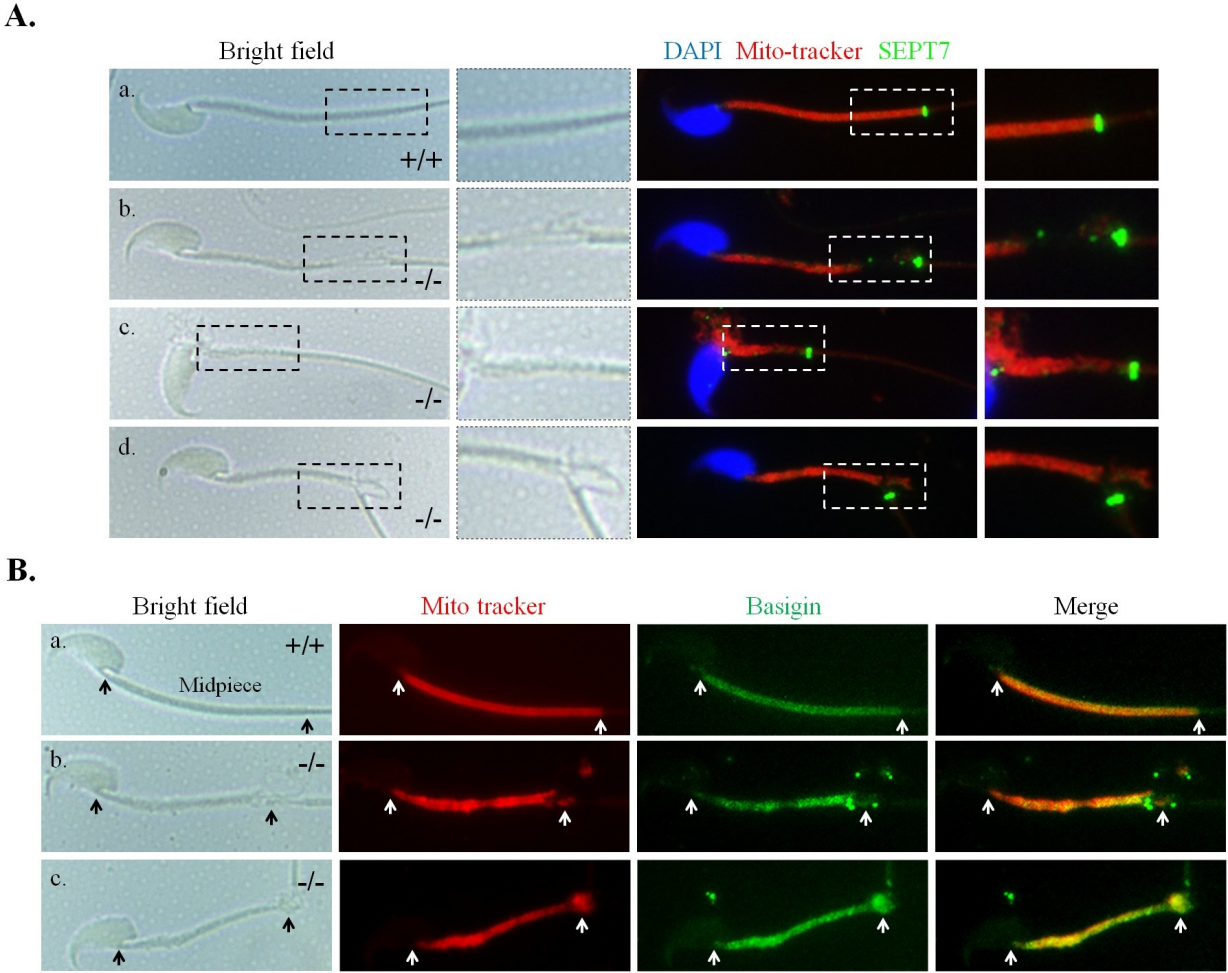
The annulus structure and function of sperm tail is intact in *Tbc1d21*^*-/-*^ mice. (A) Sperm from wild-type and *Tbc1d21*^*-/-*^ mice stained with SEPT7 antibody (green), Mito-tracker (red), and DAPI (blue). Figures from left to right indicate the bright field (Bright field), enlarged square from bright field, immune-staining figure (DAPI, Mito-tracker, and SEPT7), and enlarged square from immune-staining figure. Arrows point out the where annulus sits (SEPT7 signals, green). Figure a. for wild-type mice, and Figures b.-d. from *Tbc1d21*^*-/-*^ mice. (B) Sperm from wild-type (a.) and *Tbc1d21*^*-/-*^ (b. and c.) mice stained with Mito-tracker (red), and Basigin antibody (green). Figures from left to right indicate bright field, staining with the Mito-tracker (red), immune-staining with Basigin antibody (green) and merged figures. The length of the midpiece is indicated between the white arrows.

In Fig 1, it is shown that loss of *Tbc1d21* disrupted and decreased the length of the midpiece; however, whether this was due to the loss of the annulus structure, a critical boundary structure between the midpiece and the principal piece of the sperm tail, remains unknown. To explore the possibility, we used SEPT7 as a structural marker of the annulus. The SEPT7 signal from the sperm annulus of *Tbc1d21*^*-/-*^ mice appeared normal (Fig 2A) when compared with controls, *albeit* in the presence severe tail defects. To evaluate whether loss of *Tbc1d21* affected the annulus function, Basigin localization separated by the annulus was used as an index [25]. Basigin localization appeared within the midpiece of mature sperm, when annulus function was normal. Fig 2B shows that Basigin was preserved at the midpiece region of mature sperm from *Tbc1d21*^*-/-*^ mice, in a manner similar to that in the wild-type sperm. According to these results, we concluded that the annulus of sperm from *Tbc1d21*^*-/-*^ mice maintained structural integrity and function.

### Ultrastructural defects of the midpiece and axoneme in *Tbc1d21*^*-/-*^ sperm

**Fig 3:**
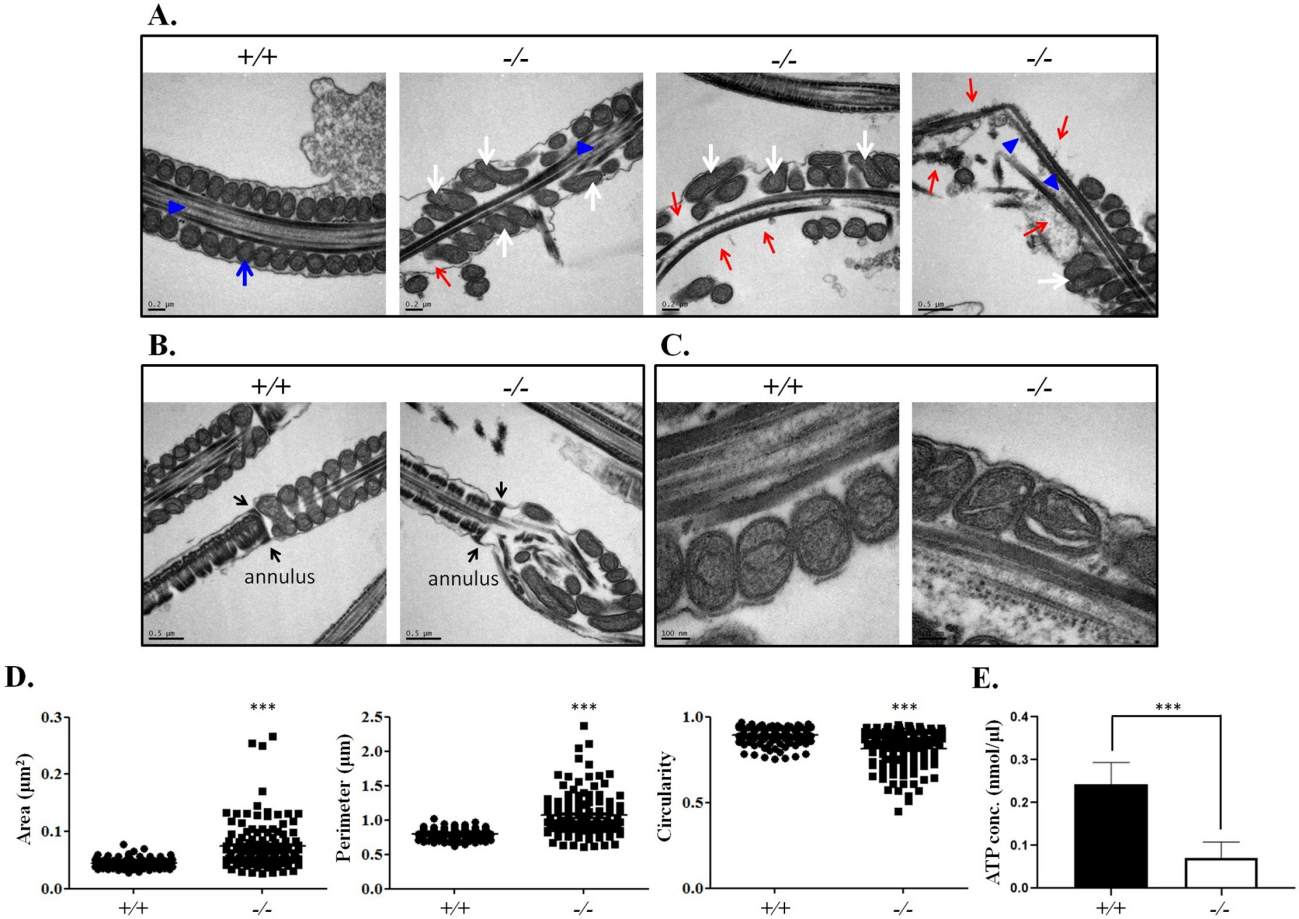
Ultrastructure of mitochondria and axoneme at wild-type and *Tbc1d21*^*-/-*^ sperm. (A-C) The midpiece structure of sperm in longitudinal section (left side) reveals the dis-localization and defects of the mitochondria and disturbed axoneme structure. (A) Blue arrows and blue arrowheads indicate the normal mitochondria and the axoneme structure in wild-type mice, respectively. In knockout mice, the abnormal mitochondria (white arrows), lacking mitochondria (red arrows), and defective axoneme structure (blue arrowheads) have been indicated. (B) Black arrows indicate the annulus structure. (C) Abnormal loss of parts of mitochondrial materials were reveled in knockout mice. (D) The statistic represents the mitochondrial defects of sperm between the wild-type and knockout mice through the evaluation of the area, perimeter, and circularity of the mitochondria. Each dot represents a mitochondrion; n > 100 per group. (E) The statistic represents the ATP levles in *Tbc1d21*^-/-^ and wild-type sperm. (***p < 0.0001, analysed using Student's *t* test).

**Fig 4:**
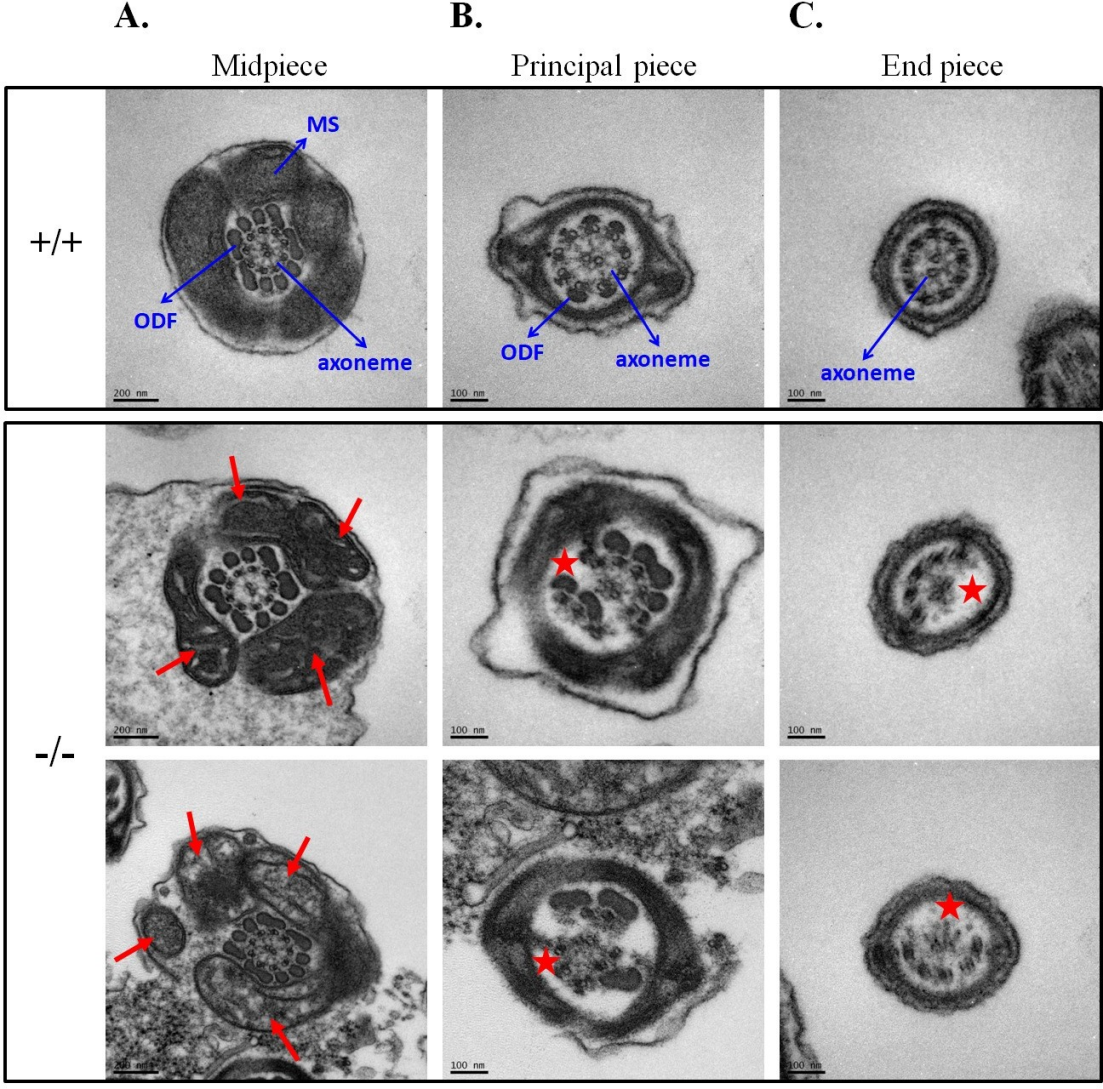
Ultrastructure of wild-type and *Tbc1d21*^*-/-*^ sperm tail cross sections. (A-C.) The midpiece (A., principle piece (B), and end piece (C) region of the sperm flagellum in cross section. The upper figures indicate the wild-type sperm, and figures at the bottom indicate the *Tbc1d21* ^-/-^ sperm tails. Mitochondria (MS), outer dense fiber (ODF), and axoneme have been indicated. Red arrows and stars indicate the defective MS and axoneme structure, respectively, in the *Tbc1d21* knockout sperm.

In order to investigate the ultrastructural defects of the sperm tail, electron microscopy (EM) was performed. Consistent with light microscope analysis, Fig 3A and 3B reveal the comprehensive ultrastructural abnormalities of mitochondria in the midpiece region of the sperm from *Tbc1d21*^*-/-*^ males with disorganized arrangement, abnormal diameters (white arrows, Fig 3A), decreased number of mitochondria (red arrows, Fig 3A), and even loss of some mitochondria materials (Fig 3C). And, consistent with the immunohistochemical analysis (Fig 2), the annulus structure was normal in *Tbc1d21*^*-/-*^ sperm (black arrows, Fig 3B). To quantify the degree of structural compromise, the area, perimeter, and circularity of the mitochondria were evaluated (n > 100, per group). Fig 3D reveals the increased areas and perimeter of the mitochondria, as well as the decreasing mitochondrial circularity in *Tbc1d21*^*-/-*^ sperm, compared to those in the wild-type. Further, the level of ATP in *Tbc1d21*^*-/-*^ sperm was evaluated. Fig 3E revels the decreased ATP level in *Tbc1d21*^*-/-*^ sperm, compared to those in the wild-type. Through a transverse section of the sperm tail, the mitochondrial sheath (MS) of the midpiece showed abnormal patterns (red arrows, Fig 4A). In addition, large parts of sperm from *Tbc1d21*^*-/-*^ males possessed disorganized outer dense fibers (ODFs) and disrupted axoneme structure (red star, Fig 4B and 4C), when compared to the wild-type. These results indicate that TBC1D21 is critical for the formation of the intact structure of mitochondria, ODFs, and the axoneme within the sperm tail.

### Loss of TBC1D21 affects its interactors, TOMM20 and DNAH7

**Fig 5:**
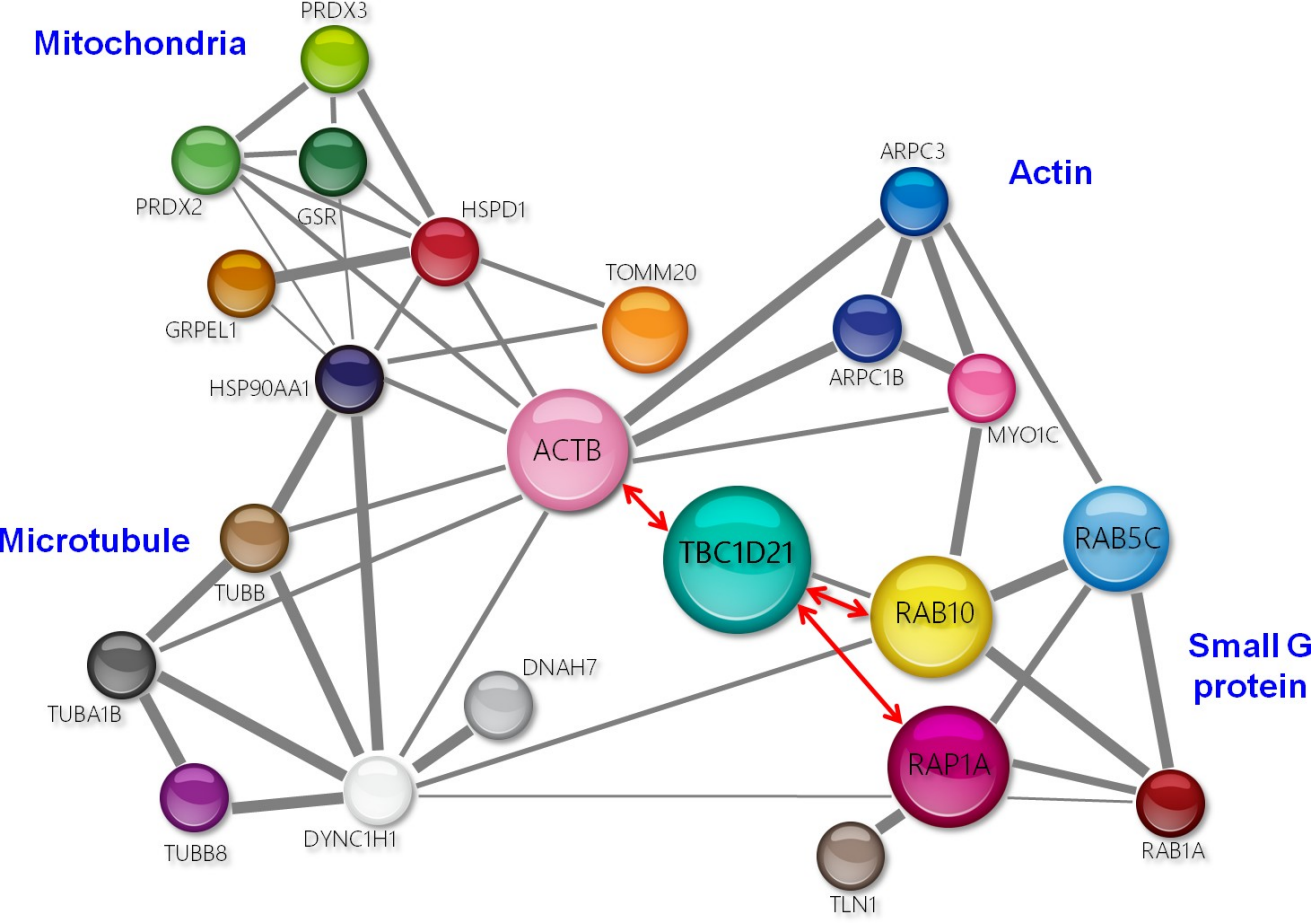
Mining and clustering the interactors of TBC1D21. The figure shows the four groups of TBC1D21 interactors as analyzed by co-IP and naao LC-MS/MS: (1) small G proteins, (2) actin-, (3) mitochondria-, and (4) microtubule-related proteins. Grey lines indicate the possible interaction between two interactors via the STRING software. Red lines present the interactions demonstrated through co-IP in our previous studies [31,32,47].

**Fig 6:**
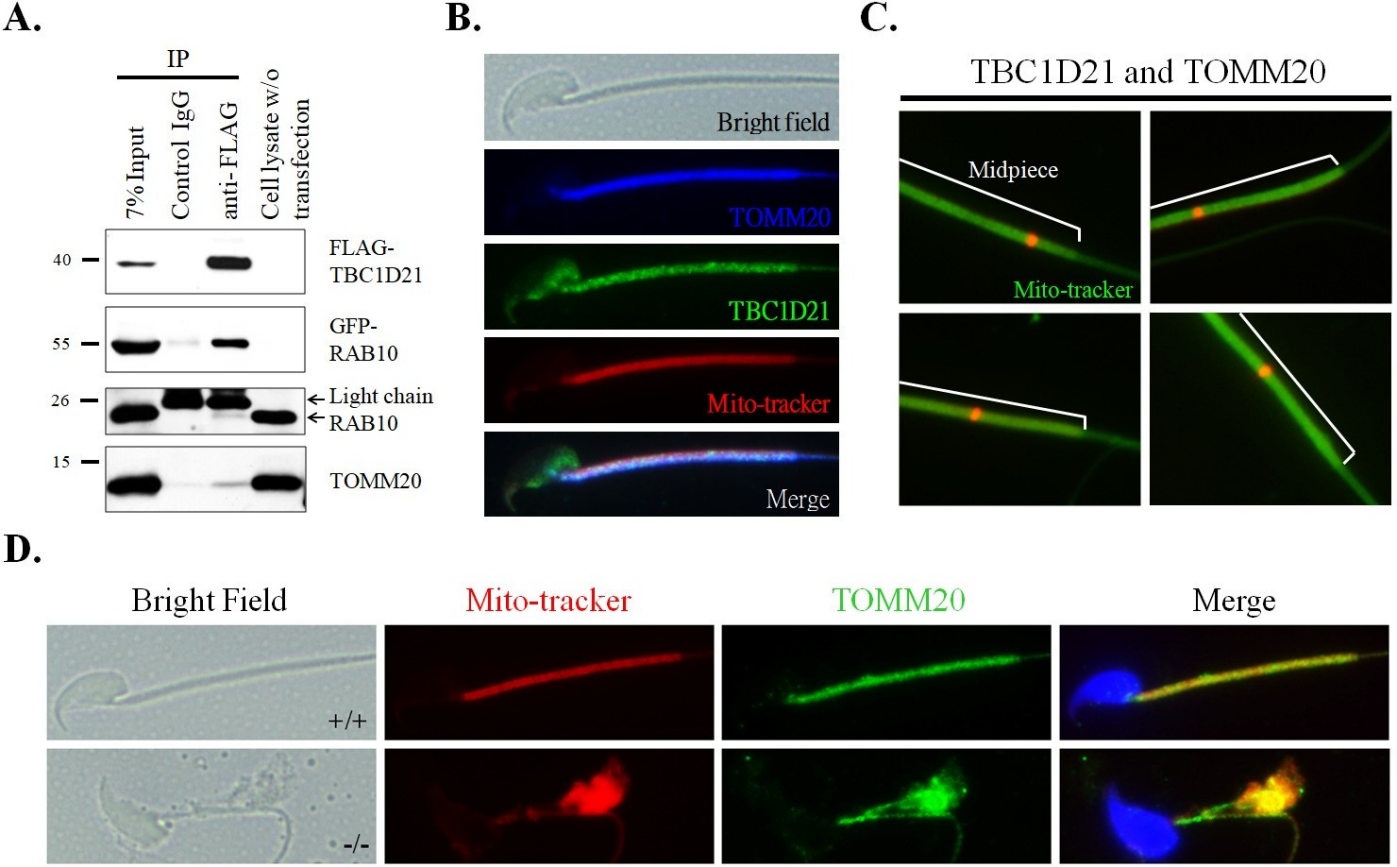
TBC1D21 interacts with one of the mitochondria membrane proteins, TOMM20. (A) co-IP of FLAG-TBC1D21 with RAB10 and TOMM20. Lysates from the cells that were transfected with the pFLAG-TBC1D21 and GFP-RAB10 vector and then immunoprecipitated with a nonspecific control IgG (Lane 2), or an anti-FLAG antibody (Lane 3), followed by immunoblotting with the FLAG antibody, GFP antibody, RAB10 antibody, or TOMM20 antibody. An input protein (7%) was used as a control during the immunoblotting of the transfected cell lysates (Lane 1). (B) Immunofluorescence detection of TOMM20 was found to be co-localized with TBC1D21 in wild-type mature spermatozoa. Figures from top to bottom indicate the bright field, TOMM20 signals (blue), TBC1D21 signals (green), Mito-tracker (red), and merged figure in wild-typmature spermatozoa. (C) Evaluation of the interaction between TBC1D21 and TOMM20 *in viv*o in four wild-type mature spermatozoa using the Duolink PLA assay. The white lines represent the midpiece region in mature spermatozoa. The arrow indicates the localization sites of the TBC1D21/TOMM20 complexes (red dots). (D) IF assay results showing disturbed localizations of TOMM20 signals in *Tbc1d21-*defective sperm, compared with wild-type sperm. Figure from left to right: the bright field, Mito-tracker (red), TOMM20 signals (green), and figure merged with Mito-tracker, TOMM20, and DAPI (blue). Figures shown as wild-type sperm (upper panels) and *Tbc1d21* knockout sperm (lower panels). Magnification = 1000X.

To define the molecular function of TBC1D21 in sperm axoneme and midpiece formation, we filtered our previously identified list of TBC1D21 interactors using the STRING web database for cellular functions related to: (1) mitochondria, (2) small G proteins, (3) actin, and (4) microtubules (Fig 5). The mitochondria-related TBC1D21 interactors could be classified into two functional types: (1) mitochondrial importing complexes for translocation of newly synthesized proteins through the mitochondrial outer membrane (e.g., TOMM20, HSP90AA1, GRPEL1, and HSPD1), and (2) anti-oxidant-related proteins (e.g., GSR, PRDX2, and PRDX3). One of the mitochondrial importing complexes, TOMM20 (translocase of outer membrane 20 of mitochondria), is located at the outer surface membrane of the mitochondria and is responsible for the recognition and translocation of cytosol-synthesized mitochondrial proteins [39–42]. To validate whether TBC1D21 interacted with TOMM20, co-IP and immunofluorescence (IF) staining were used. Fig 6A confirms that TBC1D21 interacts with RAB10 (positive control) and TOMM20 (Fig 6A). In addition, TBC1D21 co-localized with TOMM20 in the midpiece of mature sperm (Fig 6B). To further test whether there was an *in vivo* physical interaction (the distance between two proteins is approximately 40 nm) between TBC1D21 and TOMM20, proximity ligation assays were performed. In the midpiece of mature wild-type sperm, TBC1D21 definitively interacted with TOMM20, temporally (red signals, Fig 6C). Besides, loss of *Tbc1d21* revealed that TOMM20 (green signals) was disorganized and aggregated (Fig 6D and S2 Fig), instead of aligning with the sperm-tail, as in wild-type sperm.

**Fig 7:**
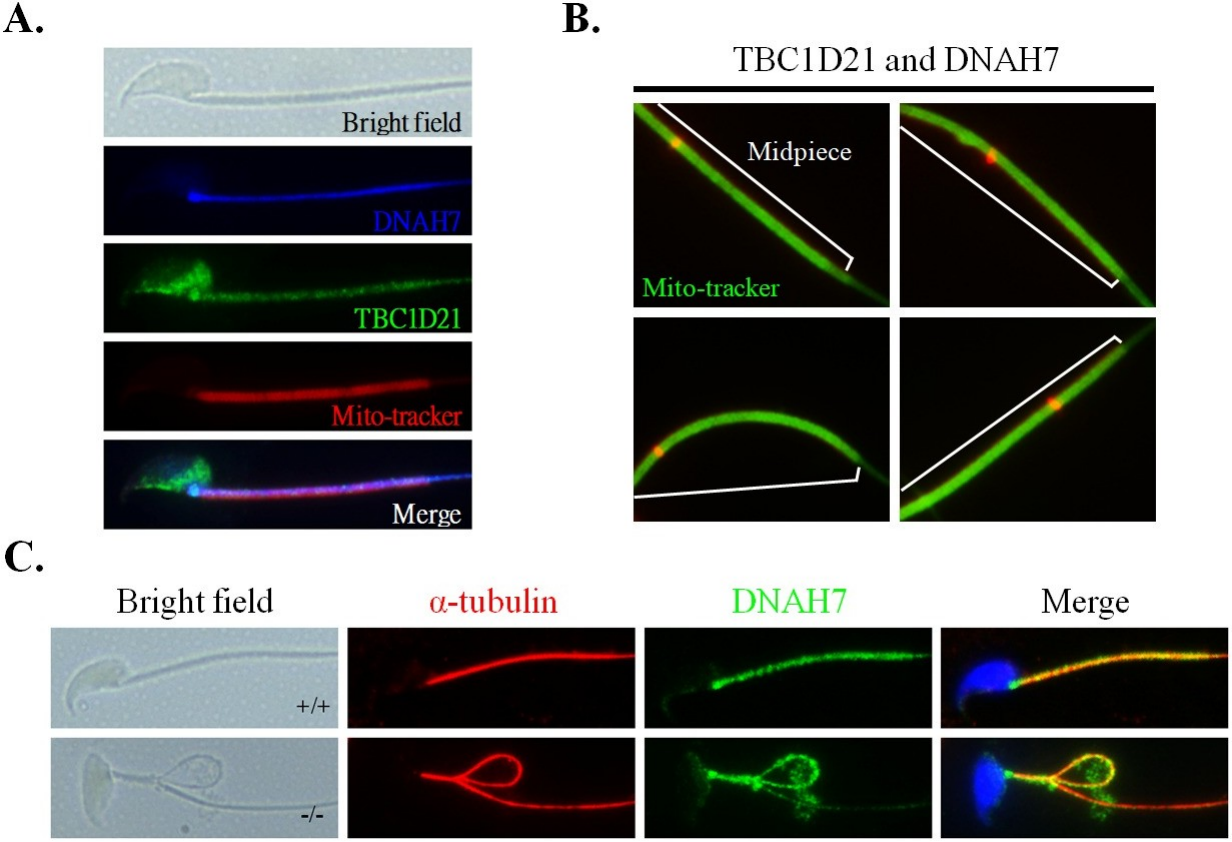
TBC1D21 interacts with one of the axoneme proteins, DNAH7. (A) Through immunofluorescence detection, DNAH7 was found to be co-localized with TBC1D21 in mature spermatozoa. Figures from top to bottom indicate the bright field, DNAH7 signals (blue), TBC1D21 signals (green), Mito-tracker (red), and merged figure in mature spermatozoa. (B) Evaluation of the protein interactions *in vivo* in four wild-type mature spermatozoa using the Duolink PLA assay. The white lines represent the midpiece region in mature spermatozoa. The arrow indicates the sites of the TBC1D21/DNAH7 complexes (red dots). (C) IF assay results show the disturbed localizations of DNAH7 signals in *Tbc1d21-*defective sperm, compared with wild-type sperm. Figure from left to right: the bright field, α-tubulin (red), DNAH7 signals (green), and figure merged withα-tubulin, DNAH7, and DAPI (blue). Magnification = 1000X.

A second putative TBC1D21 interactor was chosen for further analysis, Dynein Heavy chain 7 (DNAH7). DNAH7 is a component of the inner dynein arm of ciliary axonemes, and is highly expressed in testes [43,44]. Besides, DNAH7 signal was undetectable in the cilia from primary ciliary dyskinesia cells [43]. TBC1D21 co-localized with DNAH7 in the midpiece of the mature sperm (Fig 7A). TBC1D21 physically interacted with DNAH7 *in vivo*, as revealed via proximity ligation assays (Fig 7B, red signals). Notably, the absence of TBC1D21 resulted in the absence of DNAH7 along a subset of microtubules within the axoneme and their apparent accumulation within the residual cytoplasm (Fig 7C and S3 Fig).

Collectively, these results suggest that loss of TBC1D21 may be involved in decreasing the amount of import mitochondrial proteins as well as affecting the axoneme structure through TOMM20 and DNAH7, resulting in defects of the mitochondria and the axoneme structure of the sperm tail.

### *TBC1D21* transcripts were downregulated in sperm from teratozoospermia cases

**Fig 8:**
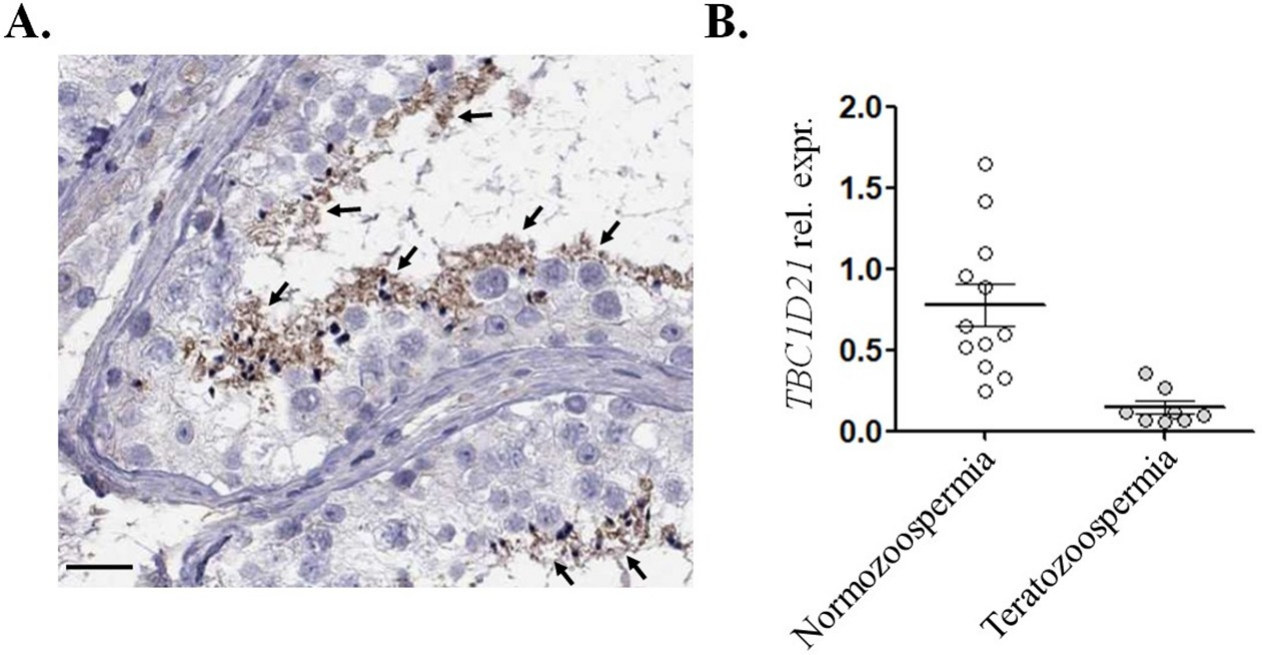
Expression of *TBC1D21* was decreased in teratozoospermia cases. (A) Immunohistochemical detection of TBC1D21 signals in the human testicular sections from The HUMAN PROTEIN ATLAS. Arrows indicate the signals on sperm and spermatids. (B) The transcript amounts of *TBC1D21* were significantly reduced in sperm samples from the teratozoospermia cases, compared with those in the fertile cases. (unpaired t-test, ***P = 0.0009).

To evaluate the clinically-relevant role of TBC1D21 in human spermatogenesis, protein localization in the human testicular tissues and RNA expression levels in teratozoospermia cases, compared with normozoospermia cases, were retrieved from The Human Protein ATLAS, as well as the published results of a cDNA microarray [38]. Fig 8A reveals that TBC1D21 was localized at elongated spermatids and mature sperm (arrows). The results from human testicular sections are similar to the murine results from our previous study. [31]. Further, the data from Fig 8B was derived from the cDNA microarray analysis of 20 sperm samples by Platts *et al*.2007 [38]. Transcript levels of *TBC1D21* in sperm were decreased in men with teratozoospermia (n = 13), compared with those in healthy controls (n = 8). These results show that TBC1D21 is significantly associated with the quantity of human sperm.

## Discussion

In our previous studies, we identified *TBC1D21* as a sterility-related GAP associated with male infertility as well as a testis-specific expressed gene [30,31]. In the present study, we have discerned the function of TBC1D21 *in vivo* through a knockout mouse model. Loss of *Tbc1d21* in mice caused male infertility, characterized by decreased sperm motility, and sperm tail defects. Lack of *Tbc1d21* resulted in a disturbed structure and utility of mitochondria, and the axoneme of the sperm tail. Two interactors of TBC1D21, TOMM20, an outer membrane subunit of mitochondria translocase, and DNAH7, an inner arm component of the axonemes, were involved, accounting for the irregular and defective patterns observed in *Tbc1d21*^*-/-*^ mouse sperm tails. From a clinical perspective, *TBC1D21* transcripts are decreased in sperm from teratozoospermia patients. Based on these results, we conclude that the cellular role of TBC1D21 is critical for the formation of intact mitochondria and axoneme of mammalian sperm.

### *Tbc1d21*-defective sperm isolated from the vas deferens had greater sperm-tail defects than those isolated from the epididymis

Fig 1D shows that the percentage of tail-defective sperms collected from the caput, cauda epididymis, or vas deferens increased gradually as we progressed from the proximal to the distal regions of the epididymis. The epididymal TBC1D21 proteins appear to play a role in this. However, the TBC1D21 protein is specifically expressed in post-meiotic male germ cells in the testis, while it is not expressed in 44 other tissue types (including those from the epididymis, seminal vesicle, and prostate among others), as indicated in the ATLAS data (https://www.proteinatlas.org/ENSG00000167139-TBC1D21/tissue) as well as in our previous study [31]. We proposed that the phenomenon occurs while the sperms pass through the epididymis. The physiological functions of the epididymis include sperm maturation, concentration, transport, immunoprotection, and storage [45]. The process of sperm maturation involves the modification of the sperm membrane proteins for increasing the flagellar beating and fertilization potential, which requires interaction of the proteins secreted from the epididymis with the male gametes [46]. We opined that flagellar beating must increase during the transport of *Tbc1d21*^-/-^sperm from the caput, cauda epididymis, to the vas deferens, and is accompanied by a gradual increase in the percentage of sperm-tail disruption. This is because the majority of structurally defective sperms have mitochondrial or axonemal irregularities.

### Irregular mitochondria and axoneme of *Tbc1d21*^*-/-*^ sperm may result from the effect on the interactors TOMM20 and DNAH7

In our previous studies, we identified interactors of TBC1D21, a RabGAP, through co-IP and nano-LC/MS [32,47]. One group of interactors consists of small GTP-binding proteins, including RAB10, RAB5C, RAB1A, and RAP1A. One of these, RAB10, showed similar localization to TBC1D21 at the manchette structure of the elongating spermatids and midpiece region of mature sperm, during murine spermiogenesis [32]. In this study, we filtered out the interactors as; (1) mitochondria-, (2) microtubule-, and (3) actin-related proteins, based on the phenotypes of the *Tbc1d21* knockout mice (Fig 5**)**. As TBC1D21 is not located within the mitochondria (unpublished data), we focused on the outer membrane proteins of the mitochondria, specifically TOMM20 from the mitochondria-related proteins. TOMM20 assists the recognition and import of mitochondrial proteins from the cytosol [39]. Loss of *Tbc1d21* also disturbed the aligning tail expression pattern of TOMM20 (Fig 6D). We propose that TBC1D21 may affect the total amount and function of mitochondrial proteins via its effect on TOMM20. Another, one of the tubulin-related proteins, DNAH7, was identified from human cilia as an inner arm component in a previous study [43]. In the current study, loss of *Tbc1d21* affected DNAH7 localization around the axoneme of the tail (Fig 7C). We suggest that parts of *Tbc1d21*^*-/-*^ sperm defects are caused by the effect on TBC1D21 interactors, TOMM20 and DNAH7. However, further biochemical and enzymatic analysis is needed to fully elucidate this mechanism.

### The possible cellular roles of TBC1D21 in intra-manchette transport during sperm-tail formation

**Fig 9:**
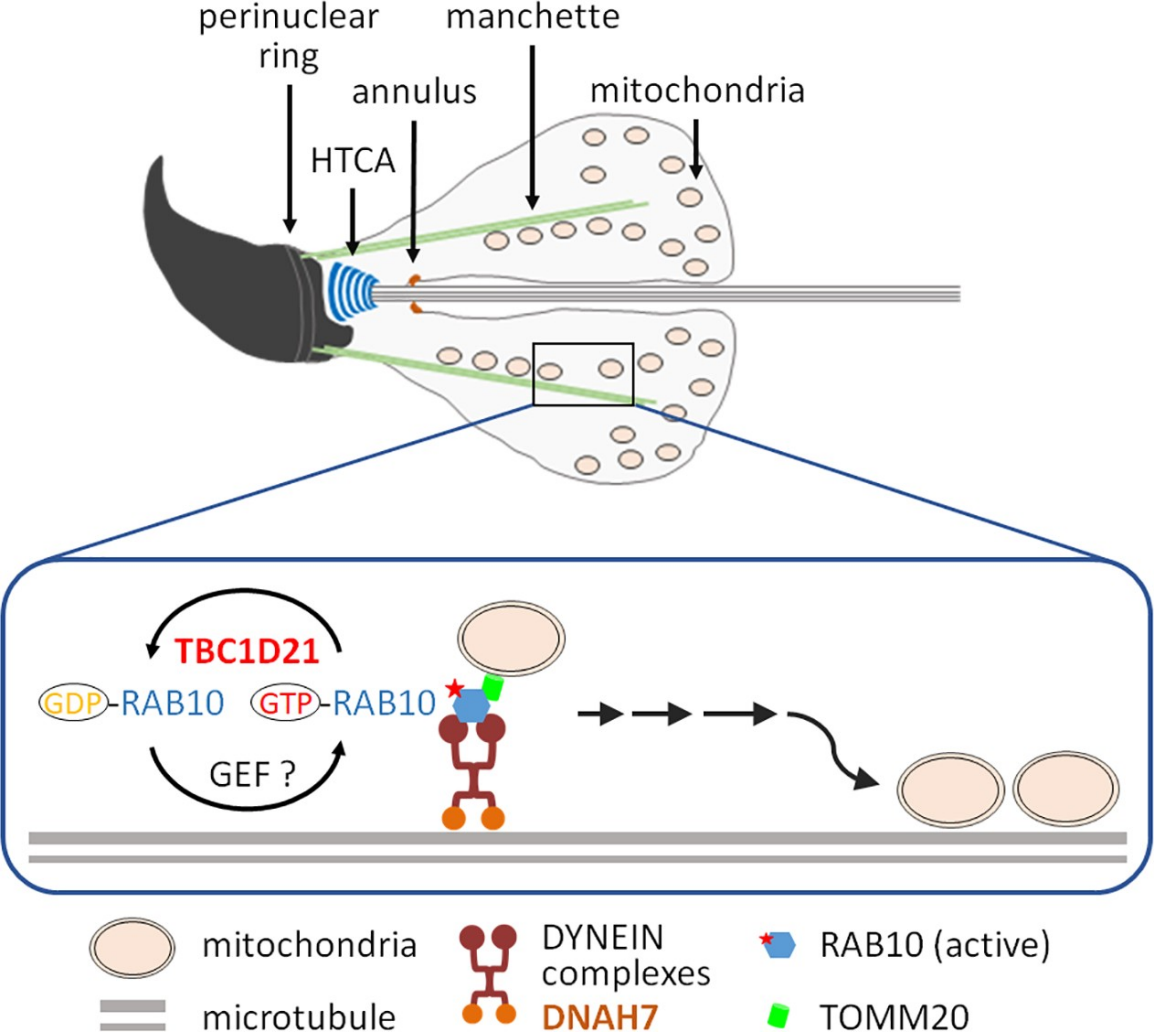
Schematic representation of the molecular function of TBC1D21 in intra-manchette transport (IMT) during sperm-tail formation. During sperm-tail formation, several components of the sperm-tail (e.g., mitochondria, fibrous sheath, and ODFs) are delivered via IMT. TBC1D21 and GEFs regulate the levels of bioactive RAB10 by facilitating conversion between activated and inactivated RAB10 (GTP- and GDP-RAB10, respectively). RAB10 acts as a vehicle for the cage (mitochondria) by interacting with TOMM20, while motility is regulated based on its interaction with the molecular motors (e.g., DYNEIN complexes) during IMT, constructed by microtubules. HTCA: head-tail coupling apparatus.

In our previous study, we found that TBC1D21 are localized to the peri-acrosomal and manchette regions in elongated spermatids and to the tail in mature sperms [31,32]. However, sperm-head defects have not been detected in *Tbc1d21*^*-*/-^ sperm; moreover, the morphology of the sperm revealed the severe defects in the mitochondria and the axoneme (Figs 3 and 4). We proposed a possible model based on our results (Fig 9). The manchette, a temporary microtubule and actin-based structure, is critical for human and murine spermiogenesis [11,48–51]. The manchette serves as platform for the transport of vesicles and proteins, which is necessary for the formation of the sperm head and tail, during intra-manchette transport (IMT) [51]. IMT plays a critical role during sperm-tail formation by facilitating the transport of mitochondria, and the components of the fibrous sheath and the ODFs, which are the two of primary structural components in the sperm tail. The RAB10-TOMM20 complexes mediate the transport of sperm-tail components (e.g., mitochondria), while motility is regulated based on its interaction with molecular motors (e.g., DYNEIN complexes) during IMT. The bioactive/inactive states of RAB10 are regulated by GEFs and TBC1D21. In TBC1D21-defective mice, the transport system (i.e., IMT) is disrupted. Additionally, observation of the spermatids revealed the severe disarrangement and loss of mitochondria, as well as the effects on the sperm-tail structure.

### Loss of TBC function causes several diseases

From 2013 to present, studies from several researchers have stated that a dysregulated TBC family expression is involved in various human diseases. For example, (1) missense mutations of *TBC1D8B* have been identified in cases of an X-linked early-onset steroid-resistant nephrotic syndrome. In podocyte cell models, mutated *TBC1D8B* results in defective trafficking pathways [52]; (2) more than 50 mutations of *TBC1D24* are now associated with a range of inherited neurological disorders, including myoclonic epilepsy, epileptic encephalopathy, and the DOORS syndrome (deafness, onychodystrophy, osteodystrophy, mental retardation, and seizures). Additionally, the cellular functions of TBC1D24 are involved in regulating the small GTP-binding protein, ARF6 [9,53,54]; (3) truncating mutations in *TBC1D23* are responsible for a form of pontocerebellar hypoplasia, by affecting dense core vesicles and lysosomal trafficking dynamics [55]; and (4) mutated *TBC1D20* has been identified in 77 families affected by the Warburg micro syndrome, which is characterized by eye, brain, and endocrine abnormalities [56]. Mice with defective *Tbc1d20* develop blindness and male infertility [56,57]. Spermatogenic defects in the mice are caused by disrupted acrosomal formation, and Sertoli cell apoptosis [58,59]. In the current study, we investigated TBC1D21, expressed in human post-meiosis male germ cells, and observed a decrease in the *TBC1D21* expression in sperm samples from teratozoospermia patients (Fig 8). However, whether dysregulation or genetic alterations of TBC1D21/*TBC1D21* are also involved in infertility in men remains unclear.

In the present study, we investigated the role of *Tbc1d21* during murine spermiogenesis *in vivo*. Its main cellular function is to provide assistance during the development and maintenance of the structure and function of mitochondria and the axoneme during sperm terminal formation. The molecular mechanism of TBC1D21 may be linked to the import of mitochondrial proteins and structural proteins of the axoneme, through TOMM20 and DNAH7, respectively. This is the first *in vivo* study directly linking sperm biology to the function of TBC1D21.

## Supporting information

S1 Fig Loss of *Tbc1d21* alleles slightly affects spermatogenesis. (A) Schematic representation of the strategy used to generate the *Tbc1d21-*defective allele. Neo, neomycin; Probe: probe for southern blotting; CU and JD, primers for amplifying the mutant allele; CU and FD primers for amplifying the wild-type allele. MESC genomic DNA was digested with EcoRI. The 21.3 kb and the 17.3 kb fragments were from the wild-type (WT) allele and targeting allele, detected through southern blotting, respectively. Mice with the targeting alleles were mated with the *Sox-2* Transgenic (Tg) mice for generating the *Tbc1d21* knockout mice. The mice were genotyped by specific primers (the mutant allele: CU and JD primers; wild-type allele: CU and FD primers). (B) The statistics show the normalised ratio of the testis, epididymis, and vas deferens weights to the body weight. Sperm count of knockout mice was slightly decreased, compared with that of the wild-type mice. (C) Compared the acrosome number though staining with Lectin and DAPI, acrosome marker (red) and nuclear DNA (blue), on the murine testicular sections of *Tbc1d21* knockout and wild-type mice. (D) Comparison of the morphological patterns of the testis and epididymis (caput and cauda) sections from wild-type (+/+) and knockout Tbc1d21 (-/-).
(TIF)

S2 Fig Results of the immunofluorescence assay depicting the disrupted localization of TOMM20 signals in *Tbc1d21-*defective sperm. Figures from left to right: bright field, Mito-tracker (red), TOMM20 signals (green), and image formed by merging the images for Mito-tracker, TOMM20, and DAPI staining (blue). Magnification: 1000X.
(TIF)

S3 Fig Results of the immunofluorescence assay depicting the disrupted localization of DNAH7 signals in *Tbc1d21-*defective sperm. Figures from left to right: bright field, α-tubulin (red), DNAH7 signals (green), and image formed by merging the images for α-tubulin, DNAH7, and DAPI staining (blue). Magnification: 1000X.
(TIF)
