# Supplementary figures and images for: Deficiency of the Tbc1d21 gene causes male infertility with morphological abnormalities of the sperm mitochondria and flagellum in mice
Source: PLoS Genet. 2020 Sep 25;16(9):e1009020. doi: 10.1371/journal.pgen.1009020 (PMC7549768; doi:10.1371/journal.pgen.1009020)

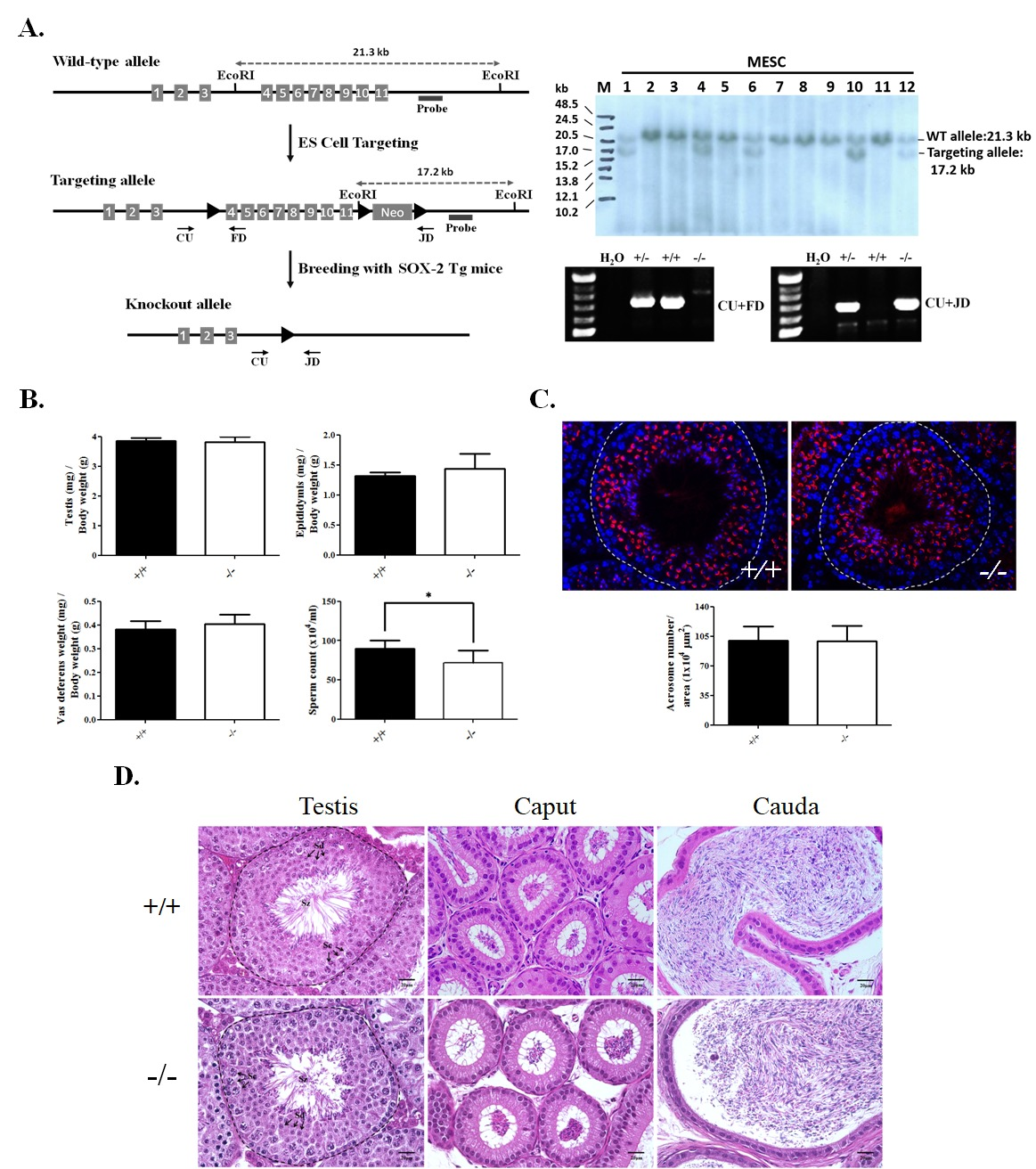

Supplement: S1 Fig — (A) Schematic representation of the strategy used to generate the Tbc1d21-defective allele. Neo, neomycin; Probe: probe for southern blotting; CU and JD, primers for amplifying the mutant allele; CU and FD primers for amplifying the wild-type allele. MESC genomic DNA was digested with EcoRI. The 21.3 kb and the 17.3 kb fragments were from the wild-type (WT) allele and targeting allele, detected through southern blotting, respectively. Mice with the targeting alleles were mated with the Sox-2 Transgenic (Tg) mice for generating the Tbc1d21 knockout mice. The mice were genotyped by specific primers (the mutant allele: CU and JD primers; wild-type allele: CU and FD primers). (B) The statistics show the normalised ratio of the testis, epididymis, and vas deferens weights to the body weight. Sperm count of knockout mice was slightly decreased, compared with that of the wild-type mice. (C) Compared the acrosome number though staining with Lectin and DAPI, acrosome marker (red) and nuclear DNA (blue), on the murine testicular sections of Tbc1d21 knockout and wild-type mice. (D) Comparison of the morphological patterns of the testis and epididymis (caput and cauda) sections from wild-type (+/+) and knockout Tbc1d21 (-/-). (TIF) [file pgen.1009020.s001.tif]

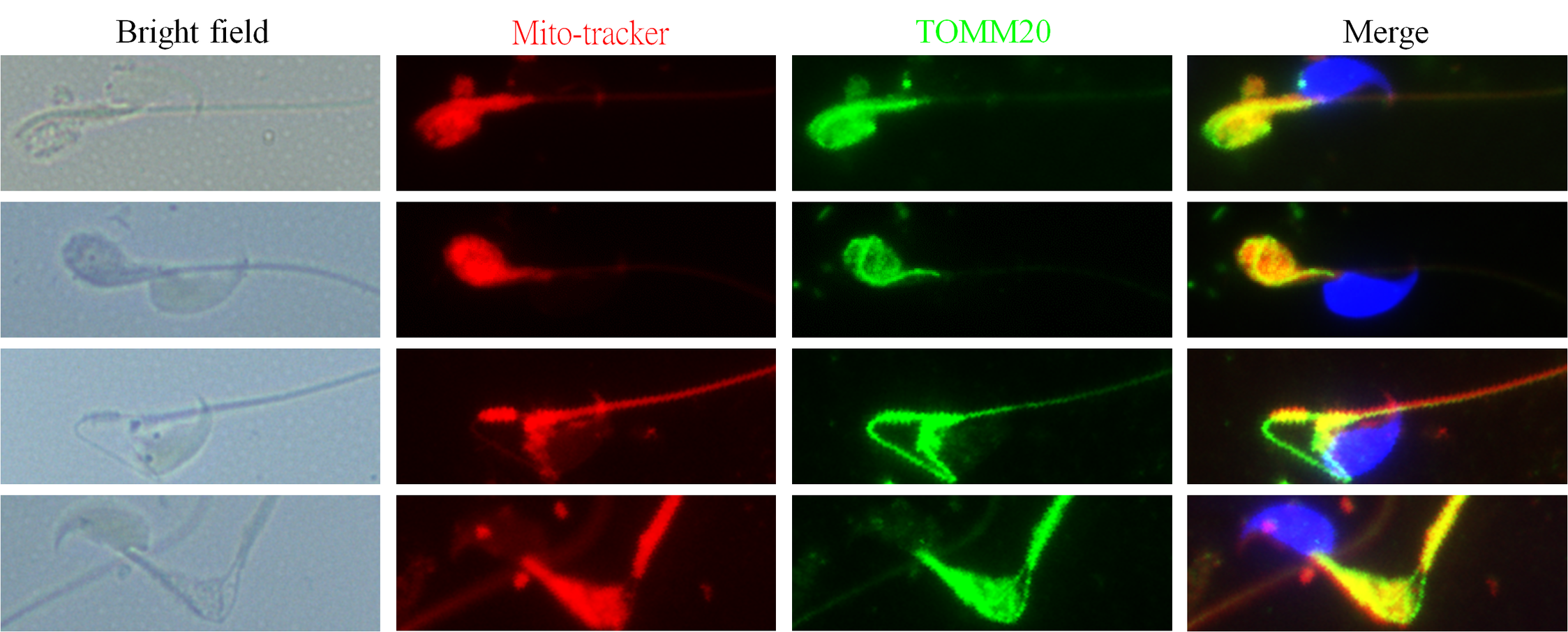

Supplement: S2 Fig — Figures from left to right: bright field, Mito-tracker (red), TOMM20 signals (green), and image formed by merging the images for Mito-tracker, TOMM20, and DAPI staining (blue). Magnification: 1000X. (TIF) [file pgen.1009020.s002.tif]

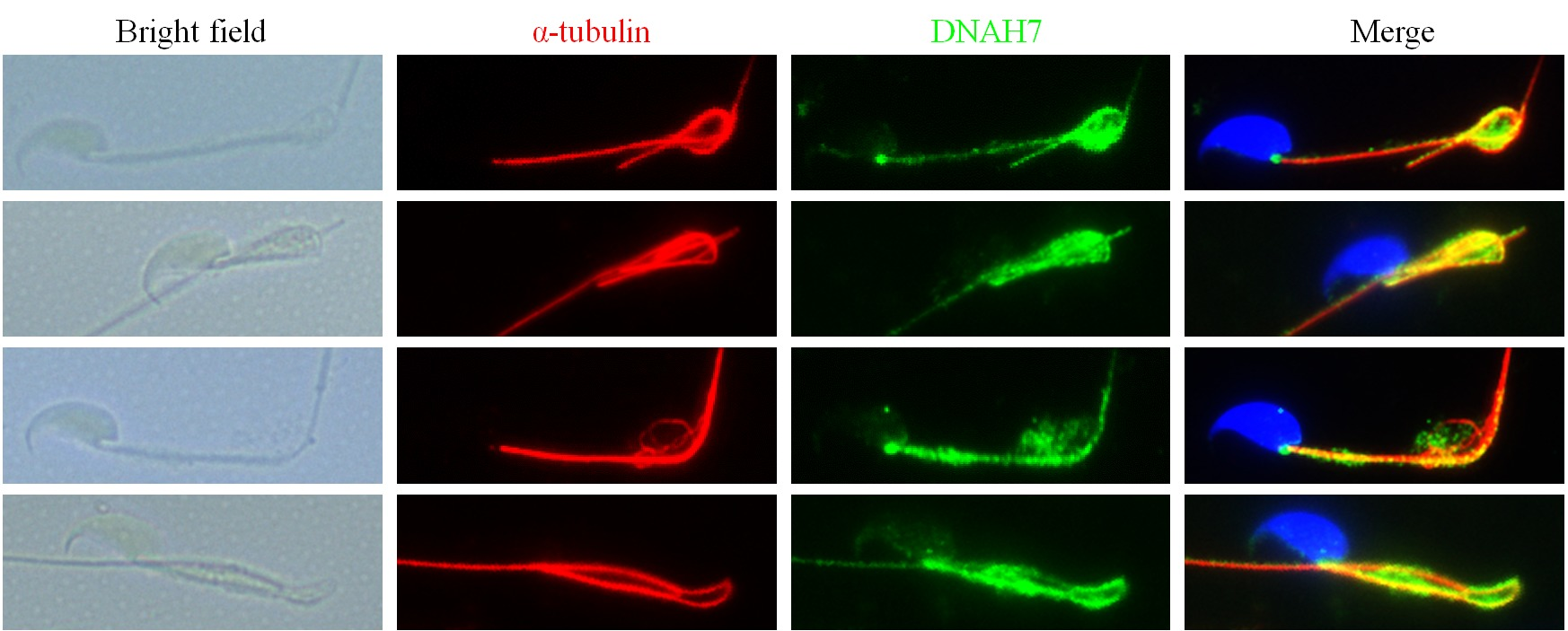

Supplement: S3 Fig — Figures from left to right: bright field, α-tubulin (red), DNAH7 signals (green), and image formed by merging the images for α-tubulin, DNAH7, and DAPI staining (blue). Magnification: 1000X. (TIF) [file pgen.1009020.s003.tif]
